# General Psychopathology, Cognition, and the Cerebral Cortex in 10-Year-Old Children: Insights From the Adolescent Brain Cognitive Development Study

**DOI:** 10.3389/fnhum.2021.781554

**Published:** 2022-01-13

**Authors:** Yash Patel, Nadine Parker, Giovanni A. Salum, Zdenka Pausova, Tomáš Paus

**Affiliations:** ^1^Institute of Medical Sciences, University of Toronto, Toronto, ON, Canada; ^2^Department of Psychiatry, Federal University of Rio Grande do Sul, Porto Alegre, Brazil; ^3^The Hospital for Sick Children, University of Toronto, Toronto, ON, Canada; ^4^Departments of Psychiatry and Neuroscience, Faculty of Medicine and Centre Hopitalier Universitaire Sainte-Justine, University of Montreal, Montreal, QC, Canada

**Keywords:** MRI, brain development, cerebral cortex, growth, cohort

## Abstract

General psychopathology and cognition are likely to have a bidirectional influence on each other. Yet, the relationship between brain structure, psychopathology, and cognition remains unclear. This brief report investigates the association between structural properties of the cerebral cortex [surface area, cortical thickness, intracortical myelination indexed by the T1w/T2w ratio, and neurite density assessed by restriction spectrum imaging (RSI)] with general psychopathology and cognition in a sample of children from the Adolescent Brain Cognitive Development (ABCD) study. Higher levels of psychopathology and lower levels of cognitive ability were associated with a smaller cortical surface area. Inter-regionally—across the cerebral cortex—the strength of association between an area and psychopathology is strongly correlated with the strength of association between an area and cognition. Taken together, structural deviations particularly observed in the cortical surface area influence both psychopathology and cognition.

## Introduction

There is overwhelming evidence demonstrating the shared heritability of psychiatric disorders (Anttila et al., 2018). Given the high rates of comorbidity (Plana-Ripoll et al., 2019), commonality in genetic and environmental risk factors (Uher and Zwicker, 2017), a transdiagnostic perspective is warranted. The “p” factor, or general psychopathology factor, is one such approach capturing latent structures of psychopathology across many disorders (Lahey et al., 2012; Caspi et al., 2014). Impairments in cognitive functioning are observed across many psychiatric disorders (Gale et al., 2010; Urfer-Parnas et al., 2010). Conceptual frameworks have suggested a bidirectional relationship between cognitive function and psychopathology (Batty et al., 2005; Calvete et al., 2013; Schweizer and Hankin, 2018). With the advent of large-scale magnetic resonance imaging (MRI) studies, group differences in the structural properties of the cerebral cortex (predominantly cortical thickness) have been reported in common psychiatric disorders (Ching et al., 2020; Hoogman et al., 2020; Thompson et al., 2020; Van den Heuvel et al., 2020; Patel et al., 2021), as well as in relation to general psychopathology (Mewton et al., 2020; Romer et al., 2021a,b) and cognitive ability (Shaw et al., 2006; Karama et al., 2014).

In this study, we investigate the association between several properties of the cerebral cortex, namely the surface area, cortical thickness, the T1w/T2w ratio (potentially an index of myelination) (Glasser and Van Essen, 2011), and neurite density [as indexed by restriction spectrum imaging (RSI)] (White et al., 2013), with general psychopathology and cognitive ability in a large sample of children from the Adolescent Brain Cognitive Development Study (ABCD) (Casey et al., 2018).

## Materials and Methods

Magnetic resonance imaging data (T1-weighted, T2-weighted, and diffusion tensor imaging) from the ABCD study of 11,753 children (mean age, 9.9 years; 48% female) were acquired and processed as described previously (Casey et al., 2018). For twin pairs, only one twin was selected at random to assess unrelated individuals only. Following quality control of the FreeSurfer pipeline (Fischl, 2012) (as described in the ABCD white papers) and removing outliers based on three times the standard deviation, there were 8,869, 8,885, 8,474, and 8,301 participants for the cortical area, thickness, T1w/T2w, and neurite density, respectively. The ABCD study conducted manual quality control of the FreeSurfer cortical surface reconstruction by scoring the extent/severity of artifacts, namely motion, intensity in homogeneity, white-matter underestimation, pial overestimation, and magnetic susceptibility artifacts. Cortical measures were averaged between the two hemispheres for each of the 34 regions of the Desikan—Killiany atlas derived by FreeSurfer (Desikan et al., 2006). Cortical thickness and the surface area were estimated through the FreeSurfer cortical reconstruction pipeline (Fischl, 2012). Neurite density was estimated by RSI using the restricted normalized directional maps, indexing intracellular and directional movement of water through neurites (White et al., 2013). The T1w/T2w ratio was quantified as the ratio of T1-weighted and T2-weighted maps sampled within the cortical ribbon (detailed in ABCD white papers, and Casey et al., 2018).

A bi-factor confirmatory factor analysis on the Child Behavior Check List (parent completed) was used to extract a general psychopathology factor, and internalizing and externalizing factors using the R package “lavaan” (Rosseel, 2012). A total of 12 questions from the CBCL questionnaire were not included in the model as they occurred with very low frequency in the sample population (<1%). The model was fit using the diagonally weighted least squares estimators implemented in “lavaan.” P-factor model item loadings, model fit, and the 12 excluded questions are presented in Supplementary Tables 1–3. The comparative fit index for the bi-factor model is 0.964, which agrees with a generally accepted threshold of good model fit of >0.95. To quantify cognitive ability, a total cognitive composite score was extracted from the youth NIH Toolbox cognitive battery. NIH Total composite measure included the following cognitive tests: flanker, dimensional change card, picture sequence memory, list sorting, pattern, oral reading, and picture vocabulary (Weintraub et al., 2013).

The relationships between psychopathology and cortical measures were modeled using linear mixed effects where psychopathology and cognition were modeled as a function of fixed effects (cortical measure, age, sex, and ethnicity), and random effects for MRI serial number (due to multiple scanners used in the ABCD study). *P*-values were corrected for multiple comparisons (34 regions tested and for each of the 4 MRI modalities for a total of 136 tests) using False Discovery Rate (FDR) (Benjamini and Hochberg, 1995).

To test the presence of mediation by cognition (or psychopathology) on the relationship between the surface area and psychopathology (or cognition), we used a simple mediation framework implemented by the “mediation” R package (Imai et al., 2011; Tingley et al., 2014). Specifically, we used a similar linear mixed effects model as above, adjusting for age, sex, ethnicity, and MRI serial number to estimate the direct effect (i.e., average direct effect, ADE), indirect effect (average causal mediation effect, ACME), and the proportion of total effect mediated. Confidence intervals were estimated using quasi-Bayesian Monte Carlo approximation (Tingley et al., 2014).

## Results

**FIGURE 1:**
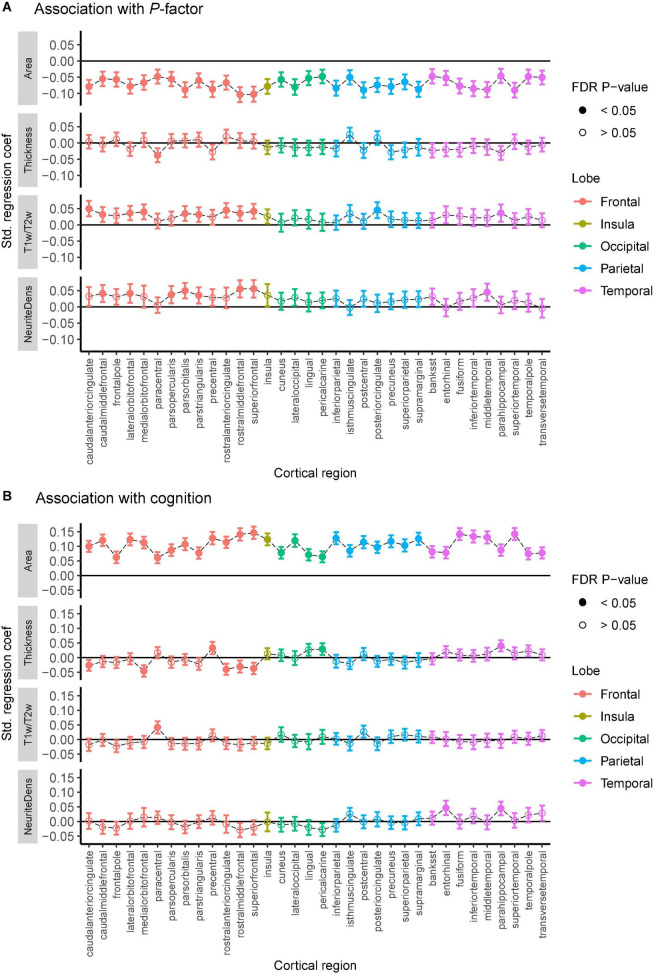
Association between general psychopathology **(A)** and cognitive score **(B)** with cortical thickness, the surface area, the T1w/T2w ratio, and neurite density (labeled “NeuriteDens”) across the 34 cortical regions of the Desikan—Killiany atlas. Standardized effect sizes (betas) plotted on the *y*-axis from linear mixed models adjusting for the effect of age, sex, and scanner effects. Error bars represent 95% confidence intervals for the estimates. Filled-in circles represent FDR-corrected *p* < 0.05.

We reveal subtle yet robust associations between cortical structure and general psychopathology and cognitive ability (Figure 1 and Supplementary Figure 1). The cortical surface area is negatively associated with psychopathology and positively associated with cognition across all cortical regions (FDR *p* < 0.05). The cortical T1w/T2w ratio and neurite density are positively associated with psychopathology, predominately in the frontal lobe. Sex-stratified analyses are reported in Supplementary Figures 2, 3. Cortical thickness is associated with cognition in eight cortical regions but shows very little association with psychopathology (Figure 1). Little to no associations are present between cognition and either T1w/T2w ratio or neurite density.

**FIGURE 2:**
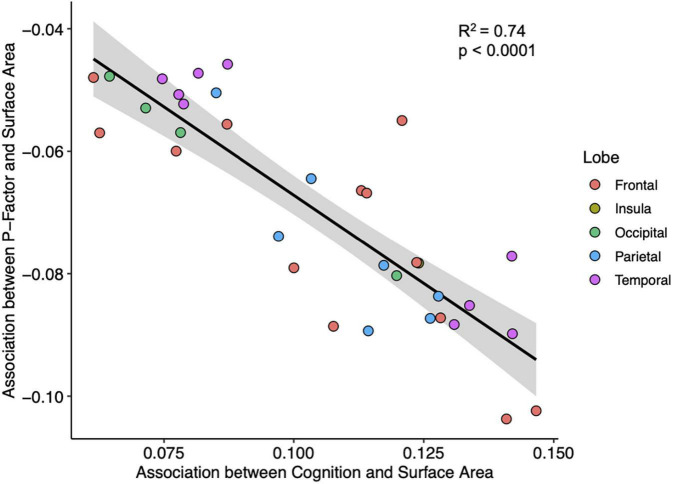
A plot of interregional associations between the surface area and cognition (*x*-axis) and interregional associations between the surface area and general psychopathology (*y*-axis) across the 34 regions of the cerebral cortex. Each point represents 1 of the 34 cortical regions. Line and shaded portions represent, respectively, linear fit and 95% confidence interval for the linear fit. Pearson correlation = −0.86, *R*^2^ = 0.74, *p* < 0.0001.

Across individuals, cognitive function is weakly correlated with psychopathology (*R*^2^ = 0.03, *p* < 0.0001). But across cortical regions, we observe a robust association between the two interregional profiles; associations between the cortical area and psychopathology correlate—across the 34 regions—with associations between the cortical area and cognitive function (*R*^2^ = 0.74, *p* = 0.0001; Figure 2). To some extent, this relationship between association-based profiles is found also with thickness, T1w/T2w ratio, and neurite density. Regression model statistics can be found in Supplementary Tables 4, 5.

Finally, we examined if cognition (or psychopathology) mediates the relationship between the surface area and psychopathology (or cognition, Supplementary Figure 4). Cognition mediates between 20 and 40% of the total effect between the surface area and psychopathology, varying across the 34 regions (Supplementary Figures 4A,B). On the other hand, psychopathology mediates between 6 and 13% of the total effect between the surface area and cognition (Supplementary Figures 4B,C). This is a tentative analysis and should be interpreted with caution as mediation analysis of cross-sectional data cannot assess the directionality of these results.

## Discussion

This report examines the relationship between multimodal measures of the cerebral cortex with cognition and general psychopathology in a large set of children from the ABCD study. There are robust associations between the radial growth of the cerebral cortex (as reflected in the cortical surface area), and both general psychopathology and cognition, as well as more subtle variations with cytoarchitectonic (neurite density) and myeloarchitectonic (T1W/TW2 ratio) features. These associations may reflect variations in developmental trajectories likely starting prenatally (the surface area) (Rakic, 1988), and continuing postnatally (intracortical myelination, dendritic branching) (Hill et al., 2010; Whitaker et al., 2016; Patel et al., 2019). It is important to note that the neurobiological underpinnings for many of these MRI-derived indices are not fully clear, and are unlikely to be specific to a single microstructural feature, such as myelin or neurite density.

In addition, there is a strong, inverse relationship between associations of the cortical area with cognition and psychopathology, respectively. Hypothetically, this may indicate an overlap between genetic (Shin et al., 2020) and environmental factors imparting—in parallel—the two behavioral phenotypes *via* the radial expansion of the cerebral cortex during prenatal development and the first few years of life. A majority of the expansion of the cerebral cortex, reflected in the surface area, occurs during prenatal and perinatal time periods (Li et al., 2013). Cross-disorder psychiatric genome-wise association studies (GWAS) point toward a role in prenatal neurodevelopment across multiple conditions, and also reveal a negative genetic correlation with GWAS of cognitive ability (Lee et al., 2019). Similarly, genetic studies of intelligence reveal the importance of neurodevelopmental processes (Savage et al., 2018). Finally, neuron progenitor specific regulatory elements are enriched with GWAS loci associated with the cortical surface area, psychiatric disorders (e.g., schizophrenia, autism, and major depression), and with intelligence and education attainment (Liang et al., 2021). Taken together, it is possible that neurodevelopment during gestation connects the processes underlying cortical growth with psychopathology and cognition. It is also possible that such early developmental events cascade into later cognitive development and psychopathology in a sequential manner (e.g., from lower cognitive abilities to higher psychopathology or *vice versa*) (Schweizer and Hankin, 2018). We have shown that a number of adverse perinatal events (e.g., hypoxia, maternal hypertension) share their molecular architecture with that underlying neurodevelopmental processes involved in cortical growth during the same period (Patel and Paus, under review).

Finally, the mediation analysis revealed differences in the amount of mediation by cognition as compared with psychopathology. This aligns with the observed lower levels of premorbid IQ in those who later develop various mental illnesses, including schizophrenia, mood disorders, substance use disorders, and any disorder, in general (Gale et al., 2010). However, it is important to note that this analysis is highly exploratory and limited by the cross-sectional nature of the analysis in this report. Temporal precedence is required *via* longitudinal data to assess directionality and ensure correct model specification (MacKinnon et al., 2007). Similarly, mediation analysis relies on strong assumptions of sequential ignorability, such that there are no unobserved covariates that influence between the independent variable (area) and the mediator, or between the mediator and the dependent variable (MacKinnon et al., 2007; Imai et al., 2010). Modeling of forthcoming longitudinal data will provide much-needed insights into the directionality of brain-psychopathology-cognition relationships, and possible strategies for modifying (unfavorable) developmental trajectories.

## Data Availability Statement

The raw data supporting the conclusions of this article will be made available by the authors, without undue reservation. The data used in the preparation of this article were obtained from the Adolescent Brain Cognitive Development SM (ABCD) Study (https://abcdstudy.org), held in the NIMH Data Archive (NDA). This is a multisite, longitudinal study designed to recruit more than 10,000 children aged 9–10 and follow them over 10 years into early adulthood.

## Ethics Statement

This study was reviewed and approved by the Hospital for Sick Children Institutional Review Board (IRB # 1000073323), and by the NIMH Data Archive (data access request ID 6959). Written informed consent to participate in this study was provided by the participants’ legal guardian/next of kin.

## Author Contributions

YP and TP conceived the research. YP wrote the first draft. GS and NP supervised the calculation of P factor. YP, NP, GS, ZP, and TP reviewed the manuscript. All authors contributed to the article and approved the submitted version.

## Conflict of Interest

The authors declare that the research was conducted in the absence of any commercial or financial relationships that could be construed as a potential conflict of interest.

## Publisher’s Note

All claims expressed in this article are solely those of the authors and do not necessarily represent those of their affiliated organizations, or those of the publisher, the editors and the reviewers. Any product that may be evaluated in this article, or claim that may be made by its manufacturer, is not guaranteed or endorsed by the publisher.
