# Supplementary material for: General Psychopathology, Cognition, and the Cerebral Cortex in 10-Year-Old Children: Insights From the Adolescent Brain Cognitive Development Study
Source: Front Hum Neurosci. 2022 Jan 13;15:781554. doi: 10.3389/fnhum.2021.781554 (PMC8823367; doi:10.3389/fnhum.2021.781554)
Supplement: Supplementary file 1 [file Data_Sheet_1.PDF]

Supplemental Content

General psychopathology, cognition and the cerebral cortex in 10-year old children: insights from the ABCD study

Yash Patel[1] PhD, Nadine Parker[1] PhD, Giovanni A Salum[2] MD/PhD, Zdenka Pausova[3] MD, Tomas Paus[1,4] MD/PhD

[1]Institute of Medical Sciences, University of Toronto, Toronto, Canada
[2]Department of Psychiatry, Federal University of Rio Grande do Sul, Porto Alegre, Brazil
[3]The Hospital for Sick Children, University of Toronto, Toronto, Canada
[4]Departments of Psychiatry and Neuroscience, Faculty of Medicine, University of Montreal, Canada

Corresponding Author:
Tomas Paus
Centre Hospitalier Universitaire Sainte-Justine, 3175 Chem. de la Côte-Sainte-Catherine, Montréal, Quebec, H3T 1C5, Canada

Supplementary Figure 1

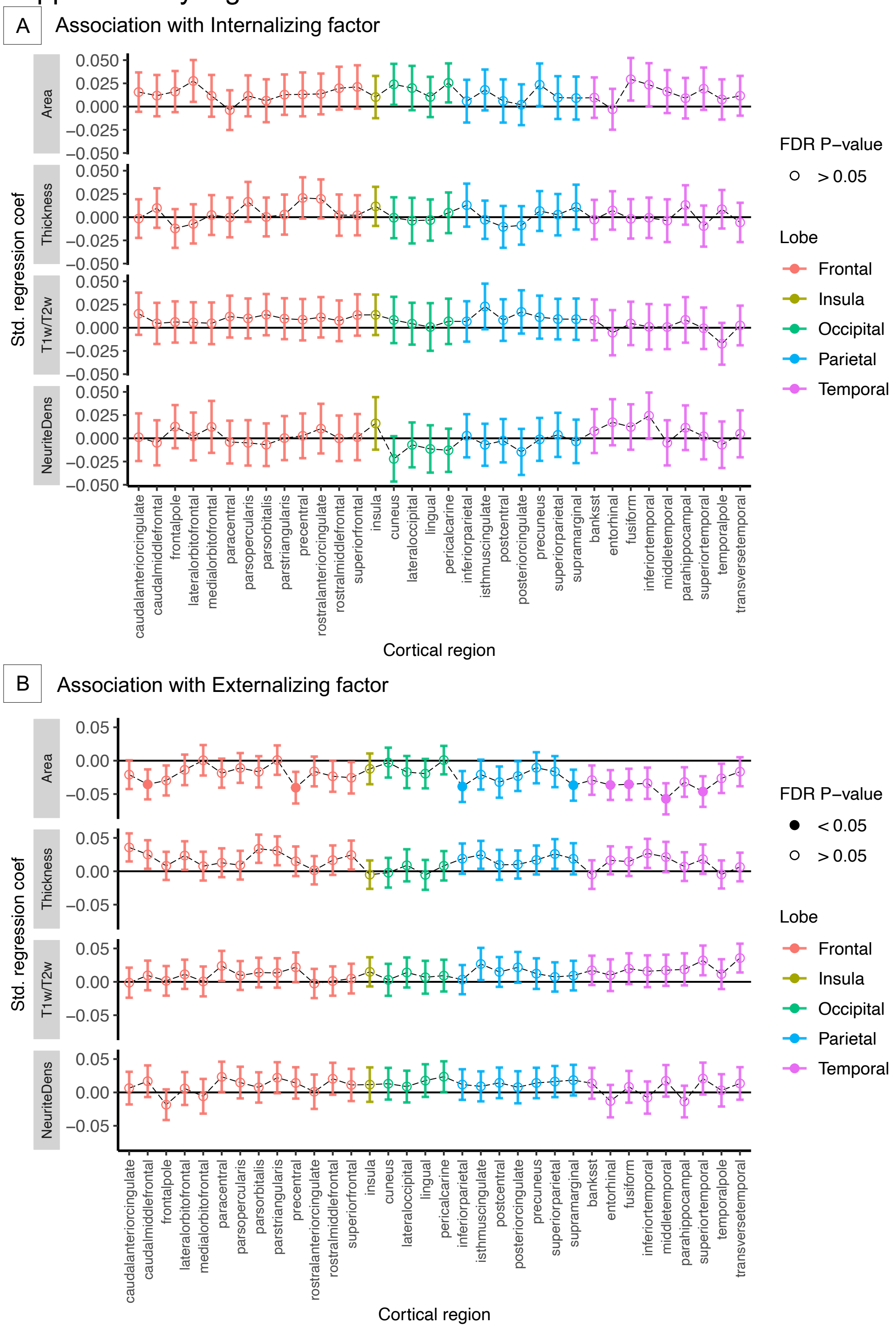

Supplementary Figure 1 Association between general internalizing (A) and externalizing factors(B) with cortical thickness, surface area, T1w/T2w ratio and neurite density (labelled "NeuriteDens") across the 34 cortical regions of the Desikan-Killiany atlas. Standardized effect sizes (betas) plotted on the y-axis from linear mixed models adjusting for the effect of age, sex and scanner effects. Error bars represent 95% confidence intervals for the estimates. Filled in circles represent FDR corrected p value < 0.05.

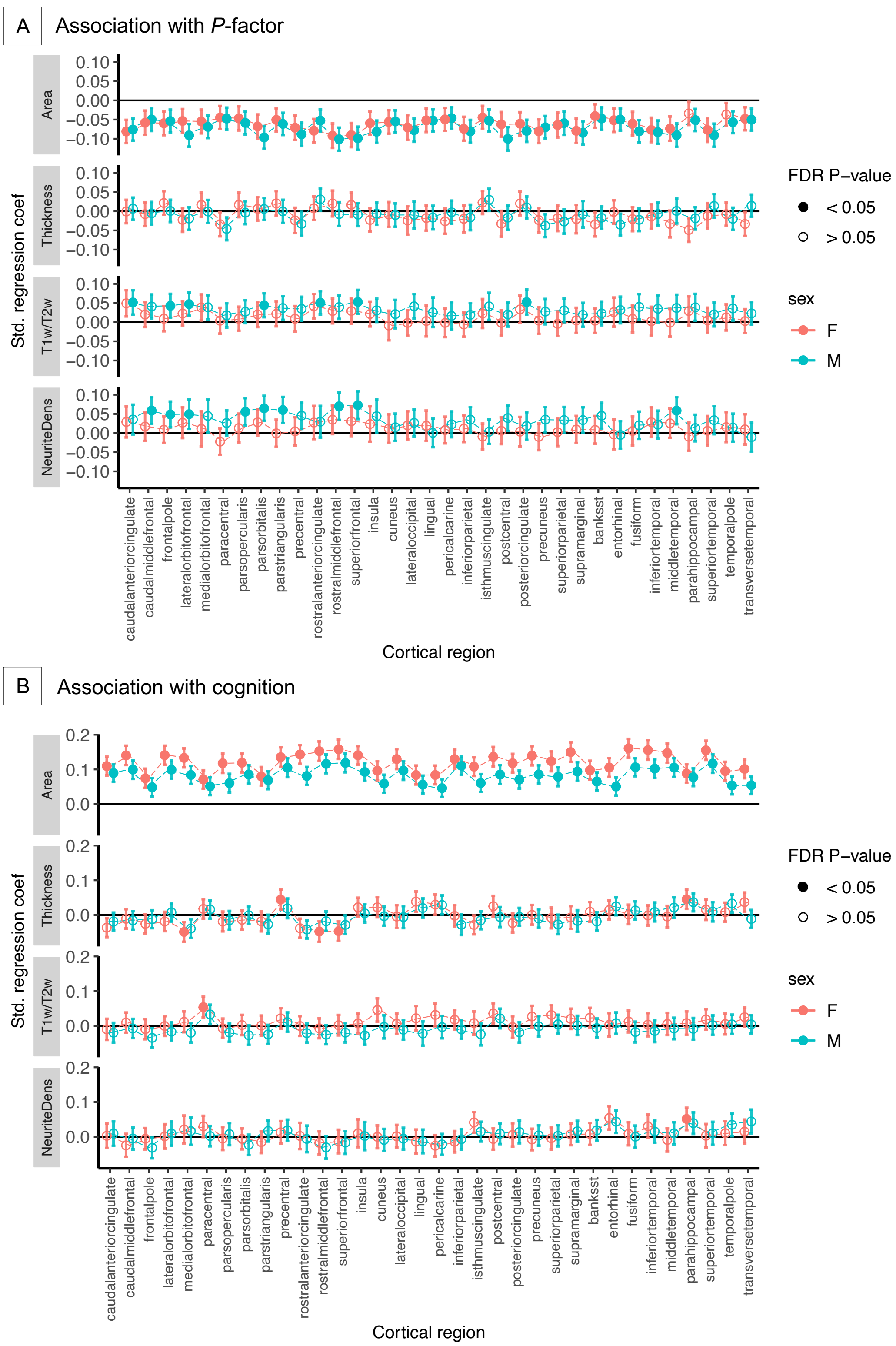

Supplementary Figure 2 Association between general psychopathology (A) and cognitive score (B) with cortical thickness, surface area, T1w/T2w ratio and neurite density (labelled “NeuriteDens”) across the 34 cortical regions of the Desikan-Killiany atlas – stratified by sex, males (M) and females (F). Standardized effect sizes (betas) plotted on the y-axis from linear mixed models adjusting for the effect of age, sex and scanner effects. Error bars represent 95% confidence intervals for the estimates. Filled in circles represent FDR corrected p value < 0.05.

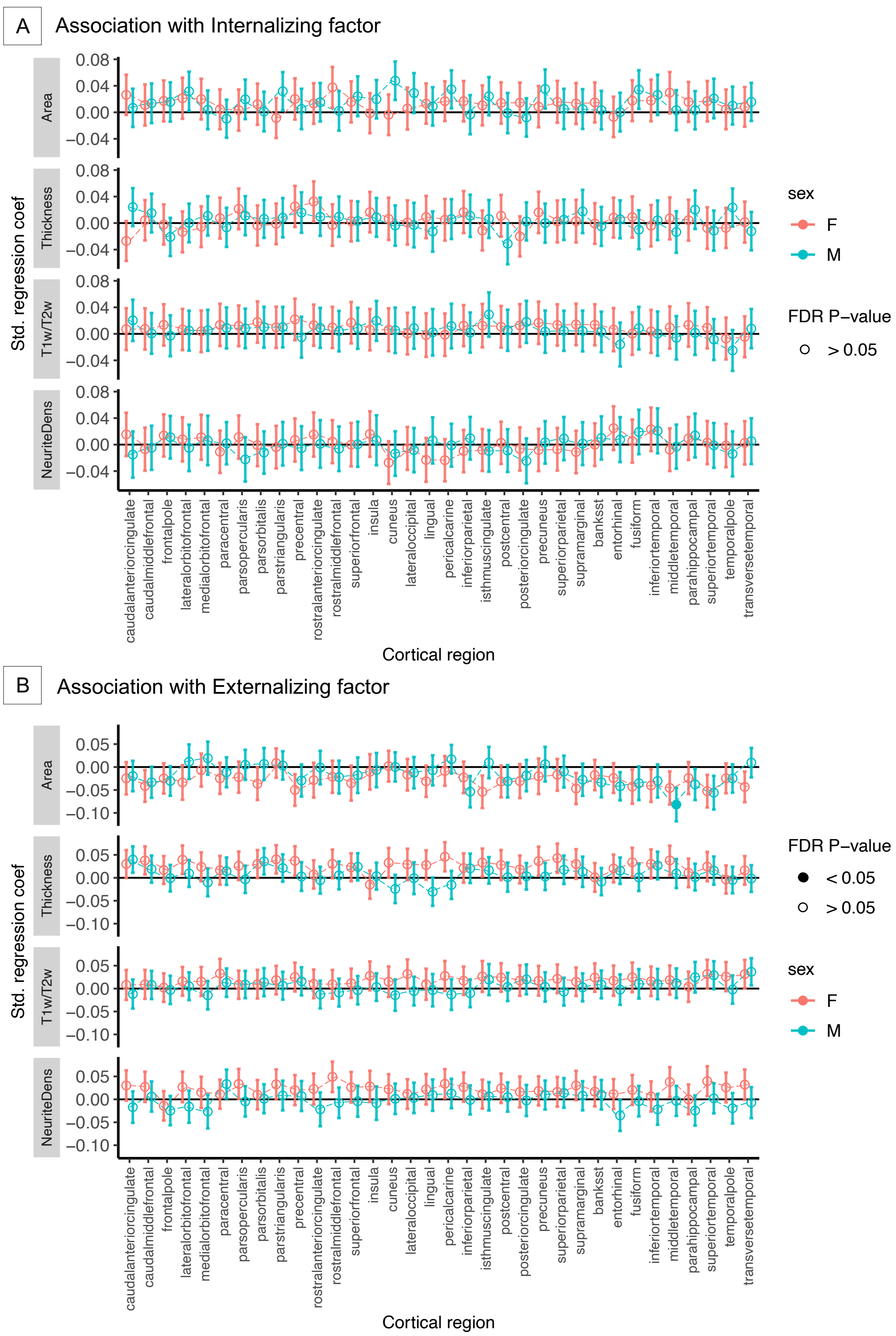

Supplementary Figure 3 Association between general internalizing (A) and externalizing factors(B) with cortical thickness, surface area, T1w/T2w ratio and neurite density (labelled “NeuriteDens”) across the 34 cortical regions of the Desikan-Killiany atlas – stratified by sex, males (M) and females (F). Standardized effect sizes (betas) plotted on the y-axis from linear mixed models adjusting for the effect of age, sex and scanner effects. Error bars represent 95% confidence intervals for the estimates. Filled in circles represent FDR corrected p value < 0.05.

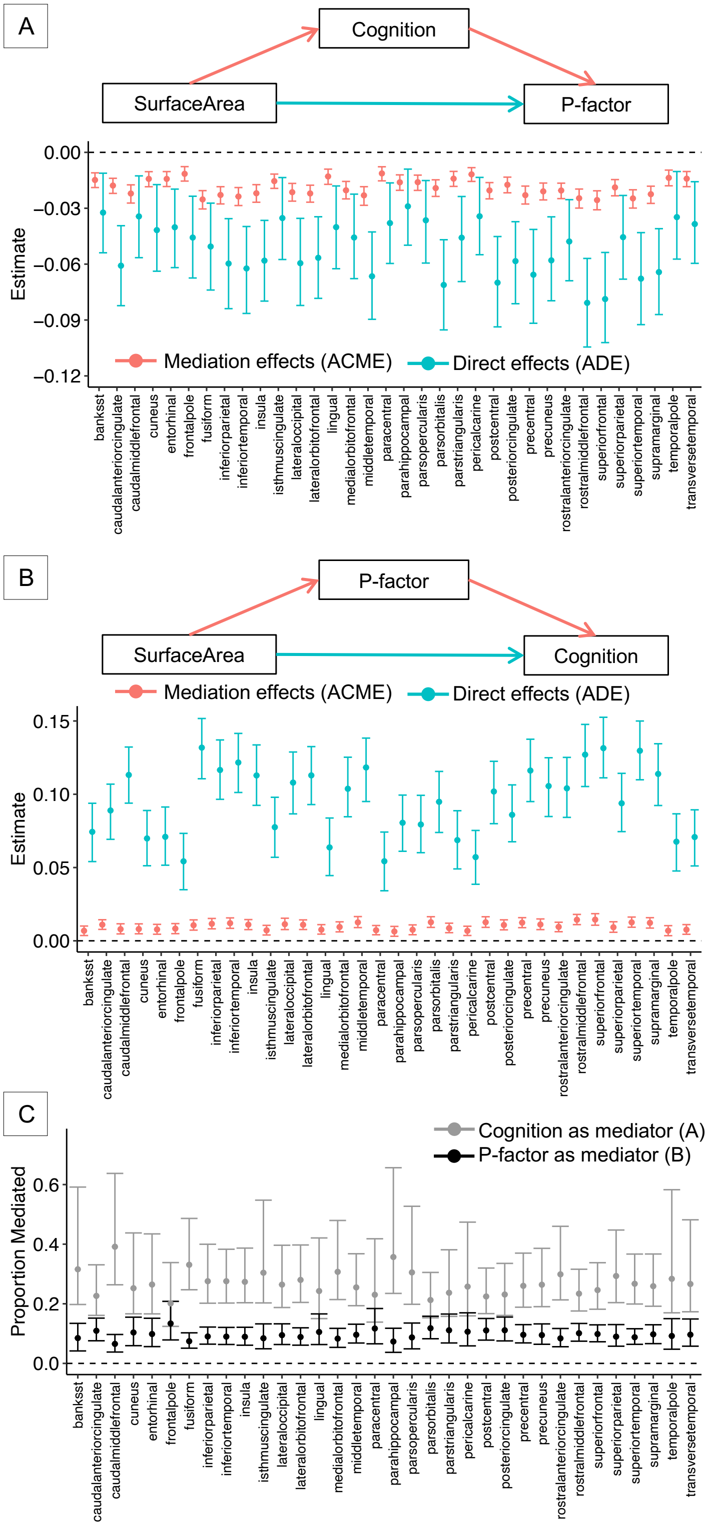

Supplemental Figure 4 Results from exploratory mediation analysis modelled for each cortical region. In A) cognition mediates the relationship between area and psychopathology (A), and in B) psychopathology mediates the relationship between area and cognition. The average direct effect (ADE) in teal represents the effect when controlling for the mediator. The average causal mediation effect (ACME) in orange represents the indirect effect going through the mediator. The proportion of the total effect that is mediated is plotted in C), for the model depicted in A) as gray, and in B) as black.
